# Use of Competitive Filamentous Fungi as an Alternative Approach for Mycotoxin Risk Reduction in Staple Cereals: State of Art and Future Perspectives

**DOI:** 10.3390/toxins11120701

**Published:** 2019-12-02

**Authors:** Sabrina Sarrocco, Antonio Mauro, Paola Battilani

**Affiliations:** 1 Department of Agriculture, Food and Environment, University of Pisa, 56124 Pisa, Italy; sabrina.sarrocco@unipi.it; 2 International Institute of Tropical Agriculture, P.O. Box 34441 Dar es Salaam, Tanzania; antomau14@libero.it; 3 Department of Sustainable Crop Production, Università Cattolica del Sacro Cuore, 29122 Piacenza, Italy

**Keywords:** beneficial filamentous fungi, *Aspergillus flavus*, *Fusarium graminearum*, *Trichoderma*, *Fusarium* Head Blight, aflatoxin, biocontrol agent, plant protection products, maize, wheat

## Abstract

Among plant fungal diseases, those affecting cereals represent a huge problem in terms of food security and safety. Cereals, such as maize and wheat, are very often targets of mycotoxigenic fungi. The limited availability of chemical plant protection products and physical methods to control mycotoxigenic fungi and to reduce food and feed mycotoxin contamination fosters alternative approaches, such as the use of beneficial fungi as an active ingredient of biological control products. Competitive interactions, including both exploitation and interference competition, between pathogenic and beneficial fungi, are generally recognized as mechanisms to control plant pathogens populations and to manage plant diseases. In the present review, two examples concerning the use of competitive beneficial filamentous fungi for the management of cereal diseases are discussed. The authors retrace the history of the well-established use of non-aflatoxigenic isolates of *Aspergillus flavus* to prevent aflatoxin contamination in maize and give an overview of the potential use of competitive beneficial filamentous fungi to manage *Fusarium* Head Blight on wheat and mitigate fusaria toxin contamination. Although important steps have been made towards the development of microorganisms as active ingredients of plant protection products, a reasoned revision of the registration rules is needed to significantly reduce the chemical based plant protection products in agriculture.

## 1. Introduction

### 1.1. Food Security and Food Safety: The Two Big Challenges towards 2050

World population may increase from 6.6 billion (in 2009) to 10.5 billion in 2050, based on a projection in 2012 by Alexandratos and Bruinsma (FAO) “World agriculture towards 2030/2050: the 2012 revision”. In some developing countries, particular some African ones, in 2050, populations are projected to be sizeable multiples of current ones, thus raising the serious issue of food (in)security because of constraints on increases in food production [1].

In this scenario, food security is one of the main challenges the world will have to confront in the next few years. At the same time, food safety, i.e., “The assurance that food will not cause harm to the consumer when it is prepared and/or eaten according to its intended use” (CODEX, 2009) is another huge issue. Food safety does not imply only the use of food not harmful for consumers but, in a more global view, it implies also the development of sustainable production tools with a low impact for the environment [2]. This is perfectly in line with the EU Directive 2009/128/EC (EU 2009/128/EC), where rules for the sustainable use of pesticides are listed in order to reduce risks and impacts on people’s health and on the environment. Furthermore, developing new tools for a sustainable agriculture perfectly fits with the public concerns constantly addressed to safe, high quality, and pesticide-free food and feed [3].

Among all the causes that can be attributed to decreasing crop productivity, yield loss due to plant pathogens plays a crucial role since plant diseases are, directly or indirectly, responsible for losses of an estimated 40 million every year [4], corresponding to 20–40% of total losses in crop yield [5].

Within the scenario of plant disease management, biocontrol based on the use of beneficial microorganisms, such as filamentous fungi, bacteria, and yeasts, is a valid and eco-friendly alternative to chemical based plant protection products. Biocontrol approaches can be used alone or as part of an integrated approach, in combination with chemical based pesticides and/or resistant cultivars resulting from breeding strategies [6].

### 1.2. Use of Beneficial Filamentous Fungi for a Sustainable Crop Protection

Starting in the 1970s, research on biological control has been intensified and much information as well as implementation of practical use has been reported [7,8]. Although biocontrol of plant disease is far from widespread, if compared with chemical based plant protection control, many commercial products containing one or more microorganisms as bioactive ingredients are currently commercially available [3,9]. In addition, new research methods, the so called “omics” approaches, are now available, thus leading to greater knowledge of the mechanisms of the actions of biocontrol agents and of their interaction with pathogens, plants, and the environment [10].

Beneficial microorganisms can interact with plant pathogens by direct or indirect mechanisms; commonly, more than one mechanism of action is involved. However, independently of the strategy used by the biocontrol agent, the result is a reduction in plant disease symptoms or toxic metabolites released, as well as an improvement in yield quantity and quality [11,12].

As regards beneficial filamentous fungi, mechanisms such as mycoparasitism, antibiosis, and competition are those which most frequently directly affect pathogen structures and activity. Instead, the induction of resistance, i.e., the stimulation of plant defense mechanisms through a cross talk involving signal molecules produced by the biocontrol agent, leads to a reduction of the disease without a direct physical contact between the biocontrol agent and the pathogen [8,13].

During mycoparasitic interactions, one living filamentous fungus can directly use another fungus as a nutrient source with necrotrophic or biotrophic parasitism. Necrotrophic (destructive) agent actions result in the death and destruction of one or more components of the host mycelium while, in biotrophic (balanced) parasitism, a living host structure favors parasite activity [14]. Necrotrophic parasites are usually more aggressive, have a wide host range, and exhibit a relatively non-specialized mode of parasitism compared to biotrophic ones. In these filamentous fungi, after the first two phases of the mycoparasitic interaction, consisting of (i) a directional growth of the antagonist towards the prey and (ii) the establishment of physical contact between the hyphae of the two fungi, the antagonistic activity is due to the production of antibiotics, toxins, or lytic enzymes that kill the pathogen [15].

The release of antibiotics and/or lytic enzymes directly affecting pathogen growth and survival-without contact between the antagonist and the pathogen- is another antagonistic strategy of beneficial fungi [16]. At the same time, the involvement of hydrolytic enzymes, also able to act synergistically with fungitoxic antibiotics, has been demonstrated in a large group of beneficial filamentous fungi, such as *Trichoderma* spp. [17,18].

The last direct mechanism used by biocontrol agents is competition for space and nutrients. In 1989, Keddy defined competition as “The negative effects which one organism has upon another by consuming, or controlling access to, a resource that is limited in availability” [19]. This definition implies two types of competition: by consumption of the resource (exploitation competition) and by controlling access to it (interference competition). Exploitation competition occurs when one organism, by exploiting the resource, reduces its availability to another organism and no contact between them is necessary. It occurs when two or more organisms have the same nutritional requirement and the organism that uses the resource more efficiently will outcompete the less efficient competitor [20]. In the case of fungi, a good competitor should have a good competitive saprotrophic ability and the ability to rapidly germinate and grow from spores [21].

Interference competition is a quite different mechanism that results in a monopolization of the habitat by antagonistic combat [19]. This kind of competition can be physical, if it involves a direct hyphal contact, or involves the production of soluble or volatile compounds for at a distance interaction with hyphal growth, or of enzymes causing the lysis of the hyphae of one fungus by another [21].

### 1.3. Mycotoxigenic Fungi: The Main Risk Affecting Cereal Production

According to a recent FAO forecast, one of the main targets of global agricultural production is staple foods, i.e., what is eaten regularly, and in such quantities as to constitute the principal part of a diet and to supply a major proportion of energy and nutrient needs. Cereals, particularly rice, wheat, and maize, represent two-third of the world’s food energy intake and are the staple food of over 4000 million people. For example, cereals represent 46% of diets, in terms of energy, of Africans, whereas in Europe, they represent 26% [22].

Cereals are the principal nutritional source for a large part of the world’s population, but they are also the target of many diseases, mostly caused by fungi, and therefore, a risk for both food security and safety. Plant pathogenic fungi are the main causes of serious diseases affecting plants [10], leading to significant reductions in yield quantity and quality, and consequently, economic losses worldwide. However, devastating plant epidemics in less developed countries frequently affect crops destined directly for human consumption and not for trade and have a social impact definitely outstripping their economic impact [23]. It is estimated that around 30% of emerging diseases are caused by fungi [24] and among this large number of specialized organisms, many are also able to produce mycotoxins, naturally occurring secondary metabolites, which, in some cases, can be extremely harmful to humans and animals, mainly by ingestion. Mycotoxins cause a variety of health problems, from acute poisoning to long-term consequences; depending on the compound, genotoxicity, carcinogenicity, immunodepression, estrogenic effects, and loss of appetite are only the main adverse effects that can be mentioned. Concern about mycotoxins and efforts aimed at minimizing consumer exposure are thus generally justifiable [25]. Regulations fixing the maximum content of mycotoxins in agricultural products, admitted or suggested, are applied almost worldwide as basic tools for consumer protection [26], and cereals, maize, and small grain, are included regarding several mycotoxins (Table 1).

Aflatoxins (AFs), mainly produced by *Aspergillus flavus* [28], are a matter of concern in maize, as for many other crops, such as nuts (peanuts, pistachio nuts), figs, almond, chili peppers, sorghum, sunflower, cotton, typically in tropical subtropical areas [29,30,31,32,33]. Recently AFs have been for the first time also reported in wild fruit in Zambia [34]. AFs may be present in many other crops/fruits not yet investigated.

Aflatoxin B_1_ (AFB_1_), classified by the International Agency for Research on Cancer (IARC) is a class 1 toxin, the highest hazard classification, confirmed carcinogenic for humans. It is also genotoxic and carry over from animals fed with contaminated feed to milk has been confirmed. Very strict limits have therefore been fixed both for food and dairy animal feed in Europe (5 μg/kg of AFB_1_), but AFs are also regulated almost everywhere. Toxins produced by *Fusarium* spp. are also among the most relevant natural contaminants in maize and wheat; fumonisins (FUMs), with *Fusarium verticillioides* and *F. proliferatum* as main producers, substantially coexist with maize, while deoxynivalenol (DON) and zearalenone (ZEN), produced mainly by *F. graminearum*, are prevalent in mild and rainy areas during maize growing. *Fusarium graminearum* is also the main actor in the Fusaria complex causing FHB of small grains.

The areas of prevalence of each mycotoxin are currently changing, due to climate change, and a wide variability in the quality and quantity of contamination has recently been reported [35,36], suggesting that mycotoxins are the most important food safety hazard affected by climate change [37]. Aflatoxins, still mentioned as a cause of death in African countries [38,39,40,41,42] are now spreading over a wider area, especially in Europe [43,44,45,46], and an increase in the risk of contamination has also been predicted in the future [47].

According to a recent survey undertaken by the BIOMIN Company on cereals and derived cereal products, DON (66%), FUMs (56%), and ZEN (53%) are the most prevalent mycotoxins in the world [48].

As regards the geographic origin of reported mycotoxin contamination, on maize, the AF/FUM mixture is the most prevalent in Africa, Asia, and South America. However, because of the movement of agricultural commodities around the globe, no region of the world is aflatoxin-free. In Europe and North America, more temperate and cold regions, mixtures of trichothecenes and a combination of trichothecenes and ZEN are the most common [29].

In the present review, two examples concerning the use of competitive beneficial filamentous fungi for the management of cereal diseases will be discussed, focusing on mycotoxins producing fungi. The authors will retrace the history of the well-established use of non-aflatoxigenic isolates of *A. flavus* to control aflatoxigenic isolates and AF contamination in maize, and will give an overview of the potential use of competitive beneficial filamentous fungi to manage FHB causal agents in wheat.

## 2. *Aspergillus flavus* and Aflatoxins in Maize

Maize (*Zea mays*) production in the period 2013–2017 was slightly above 1,000 million metric tons, the most produced cereal in the world and with about 192 million ha the second for growing area (FAOstat http://www.fao.org/faostat/en/#home). More than 60% of global production is used for feed purposes and the rest is used for food or industrial uses [49]. In Africa, maize is principally used as food and it is considered a staple, since it forms the largest percentage of calorie intake in national diets [50].

*Aspergillus flavus* is a ubiquitous fungus and is considered the main cause of AF contamination throughout the world [28]. It survives as sclerotia in soil and mycelium or sclerotia in crop debris; conidia are air dispersed and ear infection occurs after silk emergence, more efficiently at silk browning [51,52]. Damage caused by insects, such as the European corn borer (*Ostrinia nubilalis*) are reported to contribute significantly to kernel invasion and therefore AF accumulation [53,54,55,56]. Aflatoxins are commonly detected in kernels during ripening, with a rapid increase when kernel humidity drops below 30% [57]. Even if other factors than kernel water content may contribute, the final ripening period, when water activity goes below 95%, is the most suitable for rapid AF accumulation [58].

Aflatoxin production behavior varies widely between strains; strains may be able to synthetize AFB_1_ and AFB_2_ and/or cyclopiazonic acid (CPA), another mycotoxin, or completely lack production of mycotoxins. Strains that are able to produce AFs are generally called aflatoxigenic or toxigenic and strains that are not able non-aflatoxigenic or atoxigenic. Populations of *A*. *flavus* can be classified based on the size of the sclerotia produced in L-morphotype, sclerotia size 400 m, and S-morphotype, sclerotia size 400 m [59]. On average S-morphotypes are able to produce higher quantities of AFs compared to the L-morphotype [60]. Each morphotype is further classified in Vegetative Compatibility Groups (VCGs) controlled by a series of *het* loci [61,62]. Probably the vegetative compatibility system has been developed by the fungus to avoid the transmission of deleterious viruses and/or damaged genetic materials to members that do not belong to the same VCG [63,64,65,66]. Only members that belong to the same VCG can exchange genetic material after successful hyphal fusion and formation of the heterokaryon [61]. Characters are better maintained among members that belong to the same VCG compared to members that belong to different VCGs. *Aspergillus flavus* is heterothallic with one of two mating-type alleles, *MAT1-1* and *MAT1-2* carried by each single individual [67].

Twenty-five genes are involved in the biosynthesis of AFs. These genes in *A*. *flavus* are clustered within a 70-kb DNA in a subtelomeric region in chromosome III [68], and three genes encoding for the fatty acid synthase (FAS) alpha (5.8 kb) and beta (5.1 kb) subunits and the polyketide synthase (PKS; 6.6 kb) occupies around 25% of the region [69]. The AF biosynthesis genes are enclosed within a 2-kb DNA region with no specific genes (5′ end) and four genes that codify for the sugar utilization gene (3′ end) [69,70]. In the toxigenic strain of *A*. *flavus* all the 25 genes are present. Conversely, atoxigenic strains can lack the production of AFs because one or more, sometimes all, genes are missing or a single nucleotide polymorphism (SNP) in a key gene for AF biosynthesis, polyketide synthase, is present [68,71,72,73,74,75].

Similar to AF genes, CPA genes are organized in a cluster. The region of 87-kb DNA with 18 predicted genes is located beyond the AF gene cluster suggesting a physical link between the two clusters [76].

At least 16 types of AFs have been characterized [69] and AFB_1_ has been recognized as the most toxic natural compound known due its cancerogenic, immunosuppressive, and teratogenic effect on humans and animals [77]. The effects of AFs can be chronic, as a consequence of the intake of small amounts for long periods, or acute, caused by the consumption of highly contaminated food. The consumption of highly AF contaminated food can in some cases be fatal, as recently happened in Kenya and Tanzania, especially for children or immunocompromised people [38,39,40,41,42].

Aflatoxin contamination is a main concern for its impact on health, but it also poses a threat for trade. Many countries have fixed strict limits for these toxins (Table 1). It is estimated that the African continent loses in exports more than $650 million [78] every year because it is not able to meet the standards fixed by markets such as the European one.

### 2.1. Mitigation Actions

As aforementioned, AFs are mainly produced by *A*. *flavus* and *Aspergillus parasiticus* and maize is one of the crops most prone to contamination. Many strategies’ either pre- or post-harvest, have been investigated to reduce and/or prevent AF contamination in maize.

Good agricultural and management practices have been developed and they contribute to mitigate AF contamination, but they are not effective enough for safe maize production [30]. Although considerable investment and great efforts have been made to develop resistant varieties, acceptable results on large scale trials have not yet been achieved [79,80]. The effect of chemical based fungicides, with different modes of action, on mycelial growth and conidial germination of *A*. *flavus* has been evaluated in vitro and in the field. Although in vitro some fungicides totally block mycelial growth and conidial germination [81,82], in the field poor results have been reported and this approach was almost abandoned for many years. Recently, two chemical-based fungicides were tested in field trials, prothioconazole and boscalid, and they reduced *A*. *flavus* contamination at values of 75% and 56%, respectively; however, AF contamination was not considered in the study [83]. Another study considered a mixture of prothioconazole and tebuconazole for AF reduction in maize grown in north Italy. Fungicide treatment reduced the AF content, compared to the untreated maize, by 62% and 72% when applied 7 and 15 days after silking, respectively. Although the authors reported that field trials were conducted for 4 years and in two locations (eight field trials in total), only AF data for two fields have been reported [84]. Therefore, those recent studies are not sufficient to support the efficacy of chemical based fungicides in reducing AF contamination.

### 2.2. Competitive Exclusion of Aspergillus flavus

In the late 1980s, it was demonstrated that an atoxigenic strain of *A. flavus*, when co-inoculated on cotton bolts with a toxigenic strain of *A*. *flavus*, was able to significantly reduce AF content compared to cotton bolts inoculated exclusively with the toxigenic strain [85]. This experiment opened the way to a most promising and, over the years, what has been confirmed as the most effective technology in reducing pre-harvest AF contamination in crops.

*Aspergillus flavus* AF36 was the first atoxigenic *A*. *flavus* strain-based product registered worldwide for the biological control of AFs in the field. The product was registered in the USA by the Environmental Protection Agency (EPA) in 2003 [86] for use on cotton and there were subsequent amendments for use on maize, pistachio, almond, and fig [87,88]. *Aspergillus flavus* strain NRRL18543 is the active ingredient and it is atoxigenic because of a SNP, that generates a premature stop codon in the sequence, on the polyketide synthase gene of the AF biosynthesis pathway [71]. Although the strain NRRL18583 has a full and functional CPA cluster, to date no solid data have been published to demonstrate that a crop treated with the biocontrol product has a higher content of CPA compared to an untreated one. Considering that *A. flavus* AF36 has been used for more than 20 years, the first commercial field treatment, on a limited scale, was authorized by the EPA in 1996, registration released in 2003, and is still active, it is reasonable to consider that robust data on the absence of a positive correlation between the treatment and the content of CPA would have been presented.

In 2004, EPA registered also Aflaguard, another biocontrol product for AF reduction in maize and groundnuts [89]. In this case the active ingredient, strain NRRL21882 completely lacks both AF and CPA clusters [90].

Aflasafe (www.aflasafe.com) is the trade name of biocontrol products developed for African countries by the International Institute of Tropical Agriculture (IITA) in partnership with the US Department of Agriculture-Agricultural Research Service (USDA-ARS). To date, Aflasafe products have been developed, registered, and made commercially available for application in maize and groundnuts in Nigeria (2014), Kenya (2015), Senegal and The Gambia (2016), Burkina Faso (2017), Zambia (2018), Tanzania (2018), and Mozambique (2019) (R. Bandyopadhyay, personal communication, 2019). Aflasafe products are at different stages of development also in Malawi, Rwanda, Uganda, Benin, Burundi, Cameroon, Democratic Republic of Congo, Ethiopia, Mali, and Zimbabwe (www.aflasafe.com).

In Italy, the selection of Italian atoxigenic strains to use as a biocontrol of AF in maize began in 2003 [75,91,92] and the first field trials were conducted in 2012 [12]. Following positive results gained in field trials, the commercial rights of the atoxigenic *A. flavus* strain MUCL54911 [12] were acquired by Pioneer Hi-Breed Italia (now Corteva Agriscience) and used to develop a biocontrol product named AF-X1. The product has been authorized in Italy for emergency use to control AF contamination in maize, intended for feed, based on art. 53 of Regulation 1107/2009 (EC, 2009) for 4 consecutive years (2016–2019, http://www.salute.gov.it/portale/temi/p2_6.jsp?lingua=italianoid=1110area=fitosanitarimenu=autorizzazioni).

The development of atoxigenic *A. flavus* based-strain products is also in progress in Argentina [93,94], Australia [95], China [96,97], Iran [98], and Thailand [99], but also in Romania, Serbia, Pakistan, Spain, Mexico, and Costa Rica [99].

The use of atoxigenic strains of *A*. *flavus* has been demonstrated to be significantly effective in reducing AF contamination in maize fields [12,94,100,101] and in other crops [31,32,33,102]; a mean AF reduction of around 80%, but also greater than 90%, in treated fields, was reported [12,41,103]. In addition, efficacy is enhanced in conducive conditions for *A. flavus* and AF production [104]; therefore, in more challenging years, with high AF contamination, the distribution of biocontrol products based on atoxigenic strains contributes significantly to making products compliant with legislation in force [105].

Different atoxigenic *A. flavus* based biocontrol products have been developed throughout the world; all of them have in common that they are native *A. flavus* strains and have been selected in the area where they are going to be used.

### 2.3. Strain Selection Rationale

Atoxigenic strains of *A. flavus* are able to displace toxigenic strains through a mechanism called “competitive exclusion” [106]. Therefore, toxigenic strains are excluded, or at least considerably limited in their development, because of the competitiveness of the atoxigenic strains applied. The competitiveness of atoxigenic towards toxigenic strains varies significantly between strains and it requires a wide adaptation to the agroecological zones to be effective in field [107]. It happens that the most effective strain in in vitro competition trials is scarcely effective in field in reducing AF production [12,103]. Wide distribution in an agroecological zone tests the wide adaptation [108] and it is a prerequisite indicator for competitiveness in the field [41].

Therefore, strain selection is crucial to obtain an effective biocontrol agent. Native strains are naturally the best adapted to an agroecological zone; their prevalence in different years, in the same zone, further supports their fitness. In vitro preliminary results and in field confirmations must support the selection procedure, after the polyphasic confirmation of atoxigenicity [12,75,92,103].

It is a common question in the scientific community if the competitive exclusion mechanism alone can justify the strong impact of atoxigenic *A. flavus* on AF production. A recent work suggested a role of extrolites and volatiles produced by atoxigenic strains. It is an interesting topic, but a preliminary result that needs to be confirmed and better supported by scientific data [109].

### 2.4. Impact on Mycotoxins Produced by Fusaria

The field application of a living organism, atoxigenic strain of *A. flavus*, causes some concern about the effect on other microorganisms, principally mycotoxin producing ones, present in the treated crop. The main concern in maize is the effect on Fusaria population and FUM production [110]. Fumonisin content was quantified in 136 maize fields treated with Aflasafe in two states in Nigeria; FUM content in neighboring untreated control plots was not statistically different from the FUM content detected in the treated plot [41].

Similar results were also obtained in Italy. Mauro et al. reported no significant difference in FUM content between maize treated with an atoxigenic strain and an untreated control [12].

## 3. *Fusarium* Head Blight on Wheat

With a production of about 742 million tonnes (mt) over the 5-year period from 2013 to 2017 (FAOStat http://www.fao.org/faostat/en/#home), wheat is the third most important crop in terms of global production. This crop is a major source of starch and energy, as well as of other components essential or beneficial for health and nowadays included in the diet of the so called “western lifestyle” [111].

Different diseases, such as rusts (wheat stem rust, caused by *Puccinia graminis* f. sp. *tritici* Ericks and Henn; wheat stripe rust caused by *P. striiformis* Westend. f. sp. *tritici*; wheat leaf rust caused by *Puccinia triticina* Eriks) and blotches (*Zymoseptoria tritici*, *Parastagonospora nodorum*, and *Pyrenophora tritici-repentis*, causal agents of *Septoria tritici* blotch, *Septoria nodorum* blotch and tan spot, respectively) can compromise wheat production, as well as recently emerged or relatively unnoticed diseases, such as wheat blast and spot blotch [112]. However, FHB, also known as wheat scab or ear blight, is identified as one of the most serious problems in almost all the wheat growing regions in the world. *Fusarium* Head Blight is caused by a complex of fungal species, around 20, mostly belonging to *Fusarium* genus, with *F. graminearum* species complex (FGSC) and related species, such as *F. avenaceum*, *F. culmorum*, and *F. poae* [113], as the major ones associated with the disease. Other species such as *F. acuminatum*, *F. chlamydosporum*, *F. equiseti*, *F. langsethiae*, *F. sporotrichiodes*, *F. cerealis*, and *F. tricinctum* can be considered less important in the global incidence of this disease [114,115,116,117,118].

From an epidemiological point of view, cultural debris, such as wheat straw, and heads at anthesis are crucial in the disease cycle. The saprotrophic lifestyle of FHB causal agents allows the pathogens to survive on crop residues [119] in the absence of the host by developing macroconidia or, as in the case of *F. graminearum*, perithecia, where ascospores are produced. Both these asexual and sexual spores constitute the primary inoculum causing infection of wheat heads at flowering. *Fusarium* Head Blight infection is favored by long periods of high moisture or relative humidity (90%) and moderately warm temperatures (between 15 to 30 °C). If these conditions occur before, during, and after flowering, they can support inoculum production and floret infection as well as the colonization of developing grains [120,121].

Fusarium Head Blight can affect both food security and safety: the disease is not only responsible for significant yield loss—by up to 30%—and for the reduction of kernel size and weight, germination rate, and protein content, but the main concern is the risk of mycotoxin contamination of grains [122]. Trichothecenes, such as DON and its acetylated forms, nivalenol (NIV) and T2/HT2 toxins, together with ZEN are among the main mycotoxins associated with FHB in wheat. These secondary metabolites are dangerous contaminants of food and feed and affect human and animal health [123]. Trichothecenes can inhibit eukaryotic protein synthesis, thus altering polypeptide chain initiation or elongation, or can inhibit polypeptide chain termination. In addition, this class of mycotoxins affects mitochondrial protein synthesis, interacts with protein sulfhydryl groups and eventually produces free radicals that generate harmful levels of oxidative stress [124]. Harvested grain may also be contaminated with ZEN, a non-steroidal pseudo-estrogenic mycotoxin, experimentally associated with estrogenic syndromes in pigs and experimental animals [125].

Different strategies have been proposed to manage FHB. In the specific case of FHB, fungicides do not represent a winning strategy to completely control the disease and prevent mycotoxin contamination; furthermore, other approaches, such as the use of resistant cultivars as well as agronomical practices, cannot assure complete protection of the crop [3].

### Beneficial Competitive Filamentous Fungi for the Biocontrol of Fusarium Head Blight

In general terms, the use of beneficial microorganisms, such as filamentous fungi, in both biological and integrated disease management strategies, is a valid tool to confront the consequences of an exasperated and repeated use of chemical based plant protection products. Thus, biological control of *F. graminearum*, and other species involved in FHB would be a valuable addition to the available pre-harvest preventive measures like crop rotation, tillage, cultivar resistance, forecasting systems, or chemical based plant protection products that, as listed before, are often not sufficient to control FHB [126]. Despite intense research concerning the possible use of filamentous fungi as biocontrol agents against FHB, as far as we know, no commercial product, containing a competitive mold as a bioactive ingredient, is currently commercially available for the management of this disease.

From an ecological point of view, the main FHB causal agent, *F. graminearum*, is considered an r-strategist. This means that it can grow quite rapidly when simple nutrients are available and that it is a poor competitor over time, if compared with other *Fusarium* species or other fungi [121]. It is, therefore, possible to limit the pathogen’s survival and growth on residues by adding other fungi that can outcompete for substrates [3,127]. This makes the application of beneficial filamentous fungi on cultural debris a sharp strategy to reduce FHB development and to prevent the risk of mycotoxin contamination of grain.

Examples of competitive filamentous fungi that are able to access the territory previously held by the pathogen when applied on cultural residues are available, while, as far as we know, no beneficial yeasts are reported to be effective as competitors for cultural debris against FHB causal agents. Isolates of *Clonostachys rosea* and *Microsphaeropsis* spp. seem to be very promising competitive filamentous fungi, even if those belonging to *Trichoderma* genus are the most efficient to outcompete with FHB causal agents and to reduce pathogen growth and sporulation on cultural debris [128,129]. In 2005, Luongo et al. described a screening of possible wheat straw competitors isolated from crop debris, resulting in the ability of *C. rosea* to suppress sporulation of *F. graminearum* and *F. culmorum* under controlled conditions [130].

The positive effect of *C. rosea* was confirmed later, when Gilbert and Haber showed a reduction of *F. graminearum (Gibberella zeae*) perithecia development on different substrates [116]. *Clonostachys rosea* strain ACM941—isolated in Manitoba, Canada—was tested under greenhouse and field conditions exhibiting an ability to reduce the FHB index by 46% and to increase crop productivity by 7%, when compared with chemical-based fungicide treatments. In addition, a significant reduction of DON contamination in grain (up to 33%) was reported after application of this isolate onto the heads of flowering wheat [131,132].

In 2015, Schöneberg et al. demonstrated the ability of *C. rosea* to compete with *F. graminearum* (*G. zeae*) for wheat straw possession, where perithecia and ascospores were affected both when the antagonist was inoculated before and after the pathogen; in the latter case, the competition allowed a reduction of perithecia and ascospore production by 73 and 100%, respectively [133].

Certain mycoparasitic species are characterized by the ability to tolerate high levels of toxic metabolites produced by the fungal prey during interaction [134]. In addition to its mycoparasitic and saprotrophic competitive lifestyle, *C. rosea* IK726 is able to detoxify ZEN through the enzyme zearalenone lactonohydrolase (ZHD101) as part of antagonistic interaction with *F. graminearum* [135]. Since *C. rosea* genome harbors a large repertoire of putative biosynthetic gene clusters encoding a plethora of secondary metabolite synthases, secretion of antifungal metabolites combined with tolerance to xenobiotics was suggested as one of the principal modes of *C. rosea* antagonism against *Fusarium* spp. [136].

When transcriptomic analyses were performed with the aim to better understand the underlying mechanisms resulting in the successful biocontrol activity of *C. rosea*, both common and specific gene expression was detected during interactions with *F. graminearum*. Genes encoding proteins involved in membrane transport, biosynthesis of secondary metabolites and carbohydrate-active enzymes were induced during the mycoparasitic attack as well as facilitator superfamily (MFS) transporters (54% of the induced genes), with predicted functions in drug resistance and transport of carbohydrates and small organic compounds [137].

Application of *Microsphaeropsis* spp. to crop residues in the field as post-harvest or pre-planting treatment significantly reduced the number of perithecia produced on two sampling dates, thus reducing the initial inoculum of *F. graminearum* (*G. zeae*) [128].

Concerning *Trichoderma*, literature is rich with examples illustrating the use of these fungi as competitors for cultural debris in the biocontrol of FHB. In addition to its ability to reduce *F. graminearum* and *F. culmorum* growth [138], and to control FHB development under field conditions [139], *T. gamsii* T6085 is a good competitor for natural substrates where a pathogen is growing and it reduces mycotoxin production, thus, demonstrating that good competitors not only reduce pathogen growth, but they are extremely efficient in reducing mycotoxin-associated risks [140]. Recently, the ability of this beneficial isolate to colonize wheat straw and, as a consequence, to significantly reduce *F. graminearum* growth and perithecia development was demonstrated (Sarrocco, personal communication). To follow biopesticide science evolution, and to confront the sometimes erratic effects of biocontrol products, *T. gamsii* T6085 has been tested in combination with another good competitor, *F. oxysporum* [141], in a multitrophic crop protection strategy. The combined effects of the two beneficial microorganisms resulted in a strong reduction of FHB causal agent development on natural substrates [139].

Finally, when Schoneberg et al. tested a list of *Trichoderma* isolates for their ability to reduce *G. zeae* wheat straw colonization and perithecia development, a *T. harzianum* isolate (T-22) resulted in a great reduction of up to 96% [133].

Although competition is one of the most difficult mechanisms of action to be investigated and, undoubtedly, the most fascinating in its complexity, it is also a useful instrument that beneficial filamentous fungi can use to limit FHB causal agent survival on crop residues. Controlling the decomposition process of cultural debris is a way to reduce the primary inoculum of the disease [127] with clear consequences in terms of food security (reduction of disease severity) and food safety (reduction of mycotoxin contamination) in wheat.

## 4. Conclusions and Future Perspective

Two examples of competitive beneficial filamentous fungi selected as active ingredients for microrganism based plant protection products have been reported in this review. The use of atoxigenic *A. flavus* for the competitive exclusion of toxigenic strains and the prevention of AF contamination is consolidated in several countries worldwide. In Europe, after 15-years of activity, a registered commercial product is still not available. Reports on filamentous fungi able to compete with the Fusaria complex involved in FHB, and therefore, potentially useful in fusaria-toxin mitigation, are available, but not yet switched to commercial fungicides.

The logical follow-up for the development of microbial biopesticides derives directly from the industrial approach of modern agriculture. In other words, biopesticides, using microorganisms as active ingredients, must be products suitable for industrial production and likely to be used following practices that have already been developed for chemical based plant protection products. They really represent one more tool available for farmers to reduce the synthetic chemical input in agricultural production, much requested by all stakeholders.

To date, to bring a plant protection product with a microorganism as active ingredient to the market is not easy, because it must conform to the same regulations as active chemical ingredients. Active substances undergo intensive evaluation and peer-review by Member States and the European Food Safety Authority (EFSA) before approval by the European Commission. Registration costs are very high, but the potential market of microorganism based plant protection products is extremely limited if compared with chemical based products; therefore, it is inconceivable to market consortia of microorganisms as plant protection products, given that each single tiny component of the consortium must be registered (at least in Europe) separately, at very great expense.

A reasoned revision of the registration rules is strongly to be desired, for the protection of both the environment and the population with the cost-effectiveness of the new crop protection tools.

Microorganisms that act with competition-based mechanisms require a thorough knowledge of the relationships that are established between the different factors involved in a disease (plant, pathogen, and other biotic and abiotic environmental factors). The knowledge acquired for their development may be the starting point for new approaches to plant protection and can be shifted to different patosystems. This knowledge, along with the development of the “omics” sciences, and in particular metagenomics, already allows us to glimpse a possible evolution of the system towards the use of consortia of microorganisms. These can be selected from all those that make up the microbiome [142] and that can contribute to making plants less susceptible to specific diseases [143].

Another possible alternative is to breed microbe-optimized plants that are able to recruit beneficial microorganisms from the environment [144]; if such beneficial microorganisms are not present, they can be distributed as consortia along with plants. This approach, not yet exploited, seems less prone to regulatory restrictions as such beneficial microorganism consortia should not fall under the plant protection product rules.

Starting from the “Prelude to biological control” of Baker and Snyder [145], a long road has been travelled, but a significant reduction in chemical based plant protection products in agriculture is not just around the corner. However, we can see approaching on the horizon new strategies that should enable the sustainable production of safe food for all human beings in the world.

## Figures and Tables

**Table 1:** Maximum levels of some mycotoxins occurring in maize and wheat (table modified from Eskola et al., 2019 [27]).

Mycotoxin	Food Crop	Established Levels (µg/kg)
**Codex Alimentarius Standard**		
Fumonisins (FB1+FB2)	Unprocessed maize	4000
Deoxynivalenol	Cereal grains (wheat, maize, and barley) for processing	2000
Ochratoxin A	Unprocessed wheat, barley, rye	5
**European Union: Maximum and Guidance Levels**		
Aflatoxins (total)	All cereals except maize and rice	4
	Maize and rice for processing	10
Fumonisins (FB_1_+FB_2_)	Unprocessed maize	4000
	Maize intended for direct human consumption	1000
Deoxynivalenol	Unprocessed durum wheat, oats, maize	1750
Ochratoxin A	Unprocessed cereals	5
	Cereals intended for direct human consumption	3
Zearalenone	Unprocessed cereals other than maize	100
	Unprocessed maize	350
	Cereals intended for direct human consumption	75
	Maize intended for direct human consumption	100
T-2/HT-2	Unprocessed barley and maize	200 *
	Unprocessed wheat, rye, and other cereals	100 *
	Maize intended for direct human consumption	100 *
	Other cereals intended for direct human consumption	50 *
**USA: Action and Guidance Levels**		
Aflatoxin B_1_	All food crops	20
Fumonisins (FB_1_+FB_2_+FB_3_)	Maize	4000 *
**Canada: Guidance Levels**		
Deoxynivalenol	Unprocessed soft wheat	2000 *
**Japan: Maximum and Provisional Maximum Levels**		
Aflatoxin B_1_	All food crops	10
Deoxynivalenol	Wheat	1100 **
**China: Maximum and Guidance Levels**		
Aflatoxin B_1_	Maize	20
	Wheat, barley, other cereals (no rice)	5
Deoxynivalenol	Maize, barley, wheat, other cereals	1000 *
Ochratoxin A	Cereals	5
Zearalenone	Wheat and maize	60 *

Aflatoxins (total) = AFB_1_, AFB_2_, AFG_1_, and AFG_2_, * Guidance level, ** Provisional maximum levels.
